# Population Structure and Antimicrobial Resistance in *Campylobacter jejuni* and *C. coli* Isolated from Humans with Diarrhea and from Poultry, East Africa

**DOI:** 10.3201/eid3010.231399

**Published:** 2024-10

**Authors:** Nigel P. French, Kate M. Thomas, Nelson B. Amani, Jackie Benschop, Godfrey M. Bigogo, Sarah Cleaveland, Ahmed Fayaz, Ephrasia A. Hugho, Esron D. Karimuribo, Elizabeth Kasagama, Ruth Maganga, Matayo L. Melubo, Anne C. Midwinter, Blandina T. Mmbaga, Victor V. Mosha, Fadhili I. Mshana, Peninah Munyua, John B. Ochieng, Lynn Rogers, Emmanuel Sindiyo, Emanuel S. Swai, Jennifer R. Verani, Marc-Alain Widdowson, David A. Wilkinson, Rudovick R. Kazwala, John A. Crump, Ruth N. Zadoks

**Affiliations:** Massey University, Palmerston North, New Zealand (N.P. French, J. Benschop, A. Fayaz, A.C. Midwinter, L. Rogers, D.A. Wilkinson);; Ministry for Primary Industries, Wellington, New Zealand (K.M. Thomas);; Kilimanjaro Clinical Research Institute, Kilimanjaro Christian Medical Centre, Moshi, Tanzania (N.B. Amani_,_ E.A. Hugho, E. Kasagama, M.L. Melubo, B.T. Mmbaga, V.V. Mosha, F.I. Mshana);; Centre for Global Health Research, Kenya Medical Research Institute, Kisumu, Kenya (G.M. Bigogo, J.B. Ochieng);; University of Glasgow, Glasgow, Scotland, UK (S. Cleaveland, R. Maganga, R.N. Zadoks);; Sokoine University of Agriculture, Morogoro, Tanzania (E.D. Karimuribo, R.R. Kazwala);; Kilimanjaro Christian Medical University College, Moshi (B.T. Mmbaga);; US Centers for Disease Control and Prevention, Nairobi, Kenya (P. Munyua, J.R. Verani, M.-A. Widdowson);; Nelson Mandela African Institution of Science and Technology, Arusha, Tanzania (E. Sindiyo);; Ministry of Livestock and Fisheries, Dodoma, Tanzania (E.S. Swai);; University of Otago, Dunedin, New Zealand (J.A. Crump);; University of Sydney, Sydney, New South Wales, Australia (R.N. Zadoks)

**Keywords:** antimicrobial resistance, bacteria, *Campylobacter jejuni*, *Campylobacter coli*, campylobacteriosis, poultry, humans, east Africa, Kenya, Tanzania, enteric infections, zoonoses

## Abstract

Campylobacteriosis and antimicrobial resistance (AMR) are global public health concerns. Africa is estimated to have the world’s highest incidence of campylobacteriosis and a relatively high prevalence of AMR in *Campylobacter* spp. from humans and animals. Few studies have compared *Campylobacter* spp. isolated from humans and poultry in Africa using whole-genome sequencing and antimicrobial susceptibility testing. We explored the population structure and AMR of 178 *Campylobacter* isolates from East Africa, 81 from patients with diarrhea in Kenya and 97 from 56 poultry samples in Tanzania, collected during 2006–2017. Sequence type diversity was high in both poultry and human isolates, with some sequence types in common. The estimated prevalence of multidrug resistance, defined as resistance to >3 antimicrobial classes, was higher in poultry isolates (40.9%, 95% credible interval 23.6%–59.4%) than in human isolates (2.5%, 95% credible interval 0.3%–6.8%), underlining the importance of antimicrobial stewardship in livestock systems.

*Campylobacter jejuni* and *C. coli* are causes of foodborne enteric infection worldwide (*1*). *Campylobacter* spp. are among the most frequent pathogens identified in diarrheal samples from persons in Africa, particularly in children (*2*), and among World Health Organization regions, the highest burden of campylobacteriosis is observed in the Africa Region (*1*). Lack of surveillance data hinders attempts to assess the actual burden in this setting (*2*), however, and determining whether *Campylobacter* is the causal agent of diarrhea can be difficult (*3*). *Campylobacter* spp. are increasingly recognized as associated with other conditions, including stunting (*4*).

Animals and foods of animal origin make an increasing contribution to human nutrition in low- and middle-income countries as sources of high-quality protein and micronutrients (*5*). Food of animal origin is also a source of zoonotic pathogens, including *Campylobacter* spp.; 3 systematic reviews identified poultry (*6*–*8*) as a source of *Campylobacter* spp. in Africa. In Tanzania, consumption of chicken meat was the only animal-related risk factor for human campylobacteriosis (*9*), and genetic studies demonstrate the possibility of transmission between poultry and children (*10*).

Molecular epidemiologic approaches have improved our understanding of sources of human *Campylobacter* infection and contributed to campylobacteriosis control programs in high-income countries (*11*,*12*). Earlier molecular studies used low-resolution techniques such as 7-gene multilocus sequence typing (MLST) (*11*,*12*), whereas in recent years, whole-genome sequencing (WGS) has played an increasing role in informing control strategies (*13*). To date, few studies of *Campylobacter* spp. in Africa using WGS exist (*14*,*15*), and even fewer have been conducted comparing human and poultry isolates (*16*).

The prevalence of antimicrobial resistance (AMR) is high among *C. jejuni* and *C. coli* isolated from humans (*9*,*16*), poultry (*16*–*18*), and other animals (*7*) in sub-Saharan Africa. Examining the genomic epidemiology of *Campylobacter* spp. and evidence for AMR in isolates from poultry and humans in this region is necessary. To this end, we integrated food safety research in northern Tanzania (*19*) with an established diarrheal disease etiology surveillance system in neighboring Kenya to provide detailed WGS and AMR data on *Campylobacter* spp. isolated from persons with diarrhea and from poultry in East Africa and to explore similarities and differences between isolates from humans and from chickens reared in different farming systems.

## Materials and Methods

### Study Setting and Sampling

#### Chicken Isolates from Tanzania

**Figure 1:**
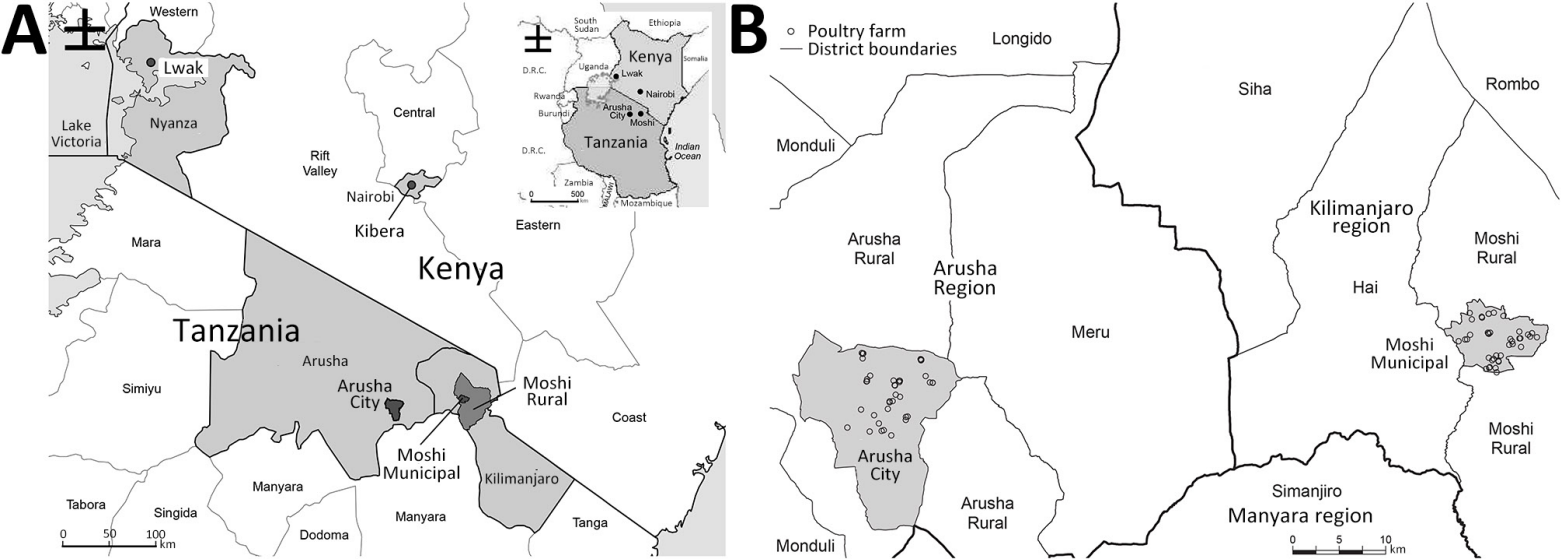
Location of sampling sites in study of population structure and antimicrobial resistance in *Campylobacter jejuni* and *C. coli* isolated from humans with diarrhea and from poultry, East Africa, 2006–2017. A) Data collection sites in Lwak and Kibera in Kenya and Arusha and Kilimanjao in northern Tanzania. Inset shows location of Kenya and Tanzania in East Africa. B) Poultry farm locations sampled in Arusha and Kilimanjaro regions, northern Tanzania.

We conducted sampling during October 10, 2016–July 24, 2017, at 66 poultry farms in Arusha City and Moshi Municipal Districts, Tanzania (Figure 1). We collected cloacal swab specimens from <10 visually healthy chickens per farm in 8 randomly selected wards in Arusha City District and 10 randomly selected wards in Moshi Municipal District. Per ward, we included up to 1 farm per production system; production systems were classified as extensive (not housed, indigenous breeds), semi-intensive (partly housed, indigenous breeds), intensive (fully housed, indigenous breeds), and broiler (fully housed, exotic breeds) (*20*). We collected cloacal swab specimens from live animals using Amies charcoal transport swabs (Sterilin Ltd, http://www.sterilin.co.uk) and transported samples in a cooler box with freezer packs to Kilimanjaro Clinical Research Institute Biotechnology Laboratory in Moshi for processing on the day of sampling. We isolated and identified *Campylobacter* as described by Sindiyo et al. (*20*) (Appendix).

#### Human Isolates from Kenya

We requested *Campylobacter* isolates collected from human stool (n = 81) from Tabitha Clinic, Kibera, Nairobi (urban informal settlement), and St. Elizabeth Lwak Mission Hospital, Asembo, western Kenya (rural site), during December 14, 2006–March 22, 2016, from the Population-Based Infectious Disease Surveillance platform, implemented by the Kenya Medical Research Institute in collaboration with the US Centers for Disease Control and Prevention (CDC) as described in Crump et al. (*19*). Isolates were shipped to Kilimanjaro Clinical Research Institute in Trypticase Soya Broth with 20% glycerol (BD Diagnostics, https://www.bd.com) and stored at −80°C (Appendix).

### Molecular Confirmation and WGS Analysis of *Campylobacter* Isolates

We sent *Campylobacter* isolates in brain–heart infusion plus glycerol on dry ice to mEpiLab, Hopkirk Research Institute, Massey University (Palmerston North, New Zealand), for WGS and analysis. Isolates were subcultured on Columbia horse blood agar (Fort Richard Laboratories, https://www.fortrichard.com) at 42°C in a microaerobic atmosphere (5% O_2_, 10% CO_2_, 85% N_2_) provided by a MACS VA500 incubator (Don Whitley Scientific, https://www.dwscientific.com). We extracted DNA using the QiaAmp DNA minikit (QIAGEN, https://www.qiagen.com) and confirmed *Campylobacter* isolates by PCR using *hipO* (*21*) and *ceuE* primers (*22*). We performed library preparation using an Illumina NexteraXT library preparation kit (Illumina, https://www.illumina.com) according to the manufacturer’s instructions. We submitted prepared libraries to New Zealand Genomics Limited (University of Otago, Dunedin, New Zealand), which performed sequencing using Illumina HiSeq 2 × 125-bp PE v4 instrument. We submitted raw sequence data to the National Center for Biotechnology Information (NCBI) (https://www.ncbi.nlm.nih.gov/bioproject) under BioProject no. PRJNA1026168, and we use accession numbers to refer to the sequences.

### Antimicrobial Susceptibility Testing and Analysis

We performed antimicrobial susceptibility testing (AST) against gentamicin, ampicillin, ciprofloxacin, nalidixic acid, erythromycin, trimethoprim/sulfamethoxazole, and tetracycline on all human and poultry isolates as described by the EUCAST disk diffusion method (*23*,*24*). We used horse blood Mueller-Hinton agar supplemented with β-nicotinamide adenine dinucleotide (Fort Richard Laboratories) with microaerobic atmosphere (MACS VA500) at 41°C for 24 +2 hours. We interpreted data according to EUCAST guidelines for *Campylobacter* for ciprofloxacin, erythromycin, and tetracycline; EUCAST guidelines for Enterobacterales for ampicillin, gentamicin, and trimethoprim/sulfamethoxazole; and Clinical and Laboratory Standards Institute guidelines for Enterobacterales for nalidixic acid (*25*). We displayed the frequencies of AMR phenotypes using UpSet plots in R (The R Project for Statistical Computing, https://www.r-project.com) using the packages ComplexUpset and ComplexHeatmap (https://github.com/krassowski/complex-upset).

We evaluated estimates of the prevalence of AMR in human and poultry isolates, where the outcome of interest was defined as resistance to >1 (AMR) or >3 (multidrug resistance [MDR]) classes, using intercept-only Bayesian regression models with AMR and MDR as Bernoulli distributed response variables. We assumed isolates from humans were statistically independent. To account for nonindependence between multiple isolates from the same flock or bird, we randomly selected 1 isolate from each farm. We repeated this random selection to create 500 random datasets, then used those datasets to create a combined posterior distribution using the outputation method (*26*). We fitted models using the R package brms (*27*) using 4 chains with 2,000 iterations per chain for each of the 500 poultry datasets and 1 × 10^6^ iterations for the human dataset and a 50% burn in. To improve convergence and avoid overfitting, we specified mildly informative, conservative priors on the fixed effects (Normal [0, 5]). We describe results as mean prevalence estimates and mean differences in prevalence between poultry and human isolates with 95% credible intervals (CrIs).

### Genetic and Phylogenetic Analyses

We characterized all isolates according to their 7-gene sequence type (ST) and clonal complex (CC) by uploading contig fasta files to the PubMLST *Campylobacter* website (*28*). In addition, we identified genes and alleles associated with resistance using the Comprehensive Antibiotic Resistance Database (*29*) and customized scripts for extracting and aligning individual genes and detecting mutations associated with resistance.

We established cgMLST allele profiles by using the 1343 gene cgMLST scheme (*30*) and plotted them as a minimum spanning tree using the MSTree V2 algorithm in GrapeTree (*31*). We created a circular dendrogram based on single linkage clustering of isolates and their cgMLST profiles with metadata on the host, farm type, *Campylobacter* species, resistome, and AST using the Interactive Tree of Life online tool (*32*).

### Ethics Statement

This study was approved by the Tanzania National Institutes for Medical Research National Research Ethics Coordinating Committee, Kilimanjaro Christian Medical University College Research Ethics Committee, the Kenya Medical Research Institute Scientific and Ethics Review Unit, the University of Otago Human Ethics Committee, and the University of Glasgow School of Veterinary Medicine Research Ethics Committee. The protocol for the source of the human isolates was approved by the Kenya Medical Research Institute Scientific and Ethics Review Committee (SSC protocol nos. 1899 and 2761). This activity was reviewed by CDC and was conducted consistent with applicable federal law and CDC policy as provided for in the Code of Federal Regulations (45 C.F.R part 46 and 21 C.F.R. part 56). Written informed consent was obtained from participants (or parent or guardian) before stool specimen collection.

## Results

### *Campylobacter* spp. Prevalence and Population Structure

We isolated *Campylobacter* spp. from 56 (8.6%) of 649 chicken cloacal swab specimens (Table). Differences in prevalence between farm types were not significant (χ^2^ test at farm level p>0.05).

**Table:** Prevalence of *Campylobacter* spp. in poultry by region and production type, Tanzania, 2016–2017*

Characteristic	Farm-level prevalence		
	Prevalence, %	No. positive	No. sampled
Region			
Arusha City	42.3	11	26
Moshi Municipal	55.0	22	40
Production type			
Extensive	37.5	6	16
Semi-intensive	43.8	7	16
Intensive indigenous	72.2	13	18
Intensive broiler	43.8	7	16

*There were no significant differences in prevalence between regions and production types (χ^2^ test p>0.6 for both contingency tables).

All isolates (n = 178) were confirmed as *C. jejuni* or *C. coli* and used for WGS and AST, including 81 from patients with diarrhea (44 from Lwak and 37 from Kibera) and 97 from 56 poultry samples (15 singletons and 82 pairs of isolates from 41 birds from 33 farms). *C. coli* made up 6 (7.4%) of 81 human isolates and 18 (18.6%) of 97 poultry isolates. The remaining isolates were *C. jejuni.*

**Figure 2:**
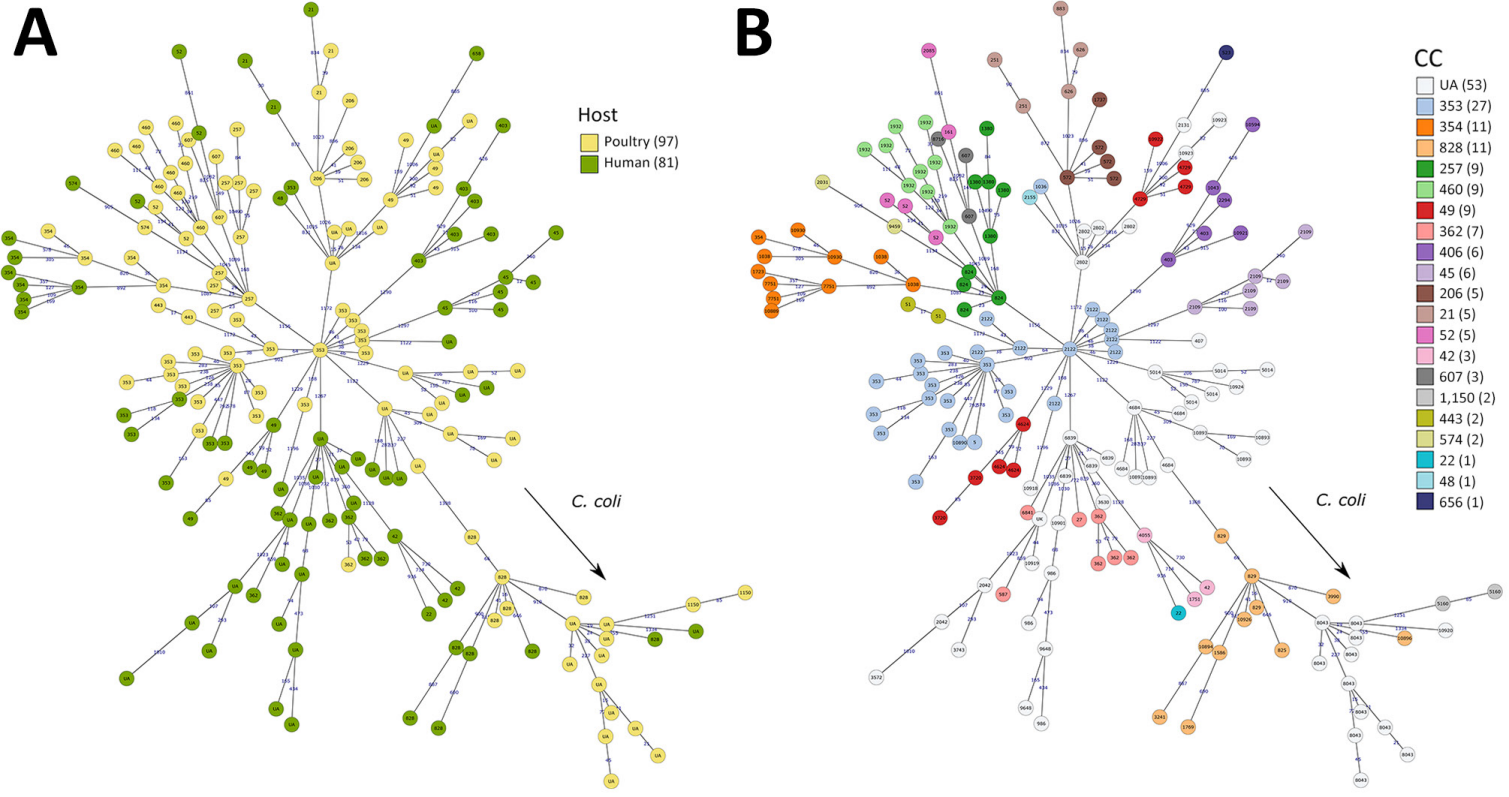
Minimum spanning tree population structure of *Campylobacter jejuni* and *C. coli* isolated from humans with diarrhea and from poultry from Kenya and Tanzania, 2006–2017 (human) or 2016–2017 (poultry), based on core genome multilocus sequence types profiles. A) Plot colored by host (human or poultry) with CC indicated in each node; B) plot colored by CC with sequence type indicated in each node. Core-genome multilocus sequence type allele differences are indicated on each branch. CC, clonal complex; UA, unassigned.

We identified 11 *C. coli* STs and 67 *C. jejuni* STs, including 57 STs from patients with diarrhea and 29 STs in poultry samples. The most common STs were *C. jejuni* ST353 (4 human and 10 poultry isolates from 6 farms), ST2122 (10 poultry isolates from 4 farms), and ST1932 (9 poultry isolates from 5 farms), and *C. coli* ST8043 (11 poultry isolates from 5 farms), each of which comprised <6.2% of the isolate collection. The most common CCs were CC353 (7 human and 20 poultry isolates), CC354 (7 human and 4 poultry isolates), and CC828 (6 human and 5 poultry isolates). Human and poultry isolates were distributed around the minimum spanning tree showing the population structure according to cgMLST (Figure 2); some clusters represented 7-gene CC or ST derived from mixed host populations and others associated with a single host (e.g., CC257, CC460/ST1932, ST2122, and ST8043 in poultry; CC45 and CC403 in humans).

By cgMLST, isolates were largely clustered according to their STs and CCs, with some exceptions. For example, *C. jejuni* CC49 isolates clustered in 2 distinct clades; 1 included ST479 and ST10922 and 1 included ST3720 and ST4624. Further, most isolates belonging to *C. jejuni* CC353 clustered together, with the exception of 1 isolate belonging to ST1036. The most closely related human and poultry isolates differed by 53 alleles on the basis of cgMLST; all were identified as ST362 based on 7-gene MLST.

At the time of writing, 22 (12.4%) isolates belonged to STs that were unique to this study (ST numbers 10893 and above). Three (1.7%) were *C. coli* isolates from CC828 and the remainder were *C. jejuni* (Appendix Tables 1, 2).

**Figure 3:**
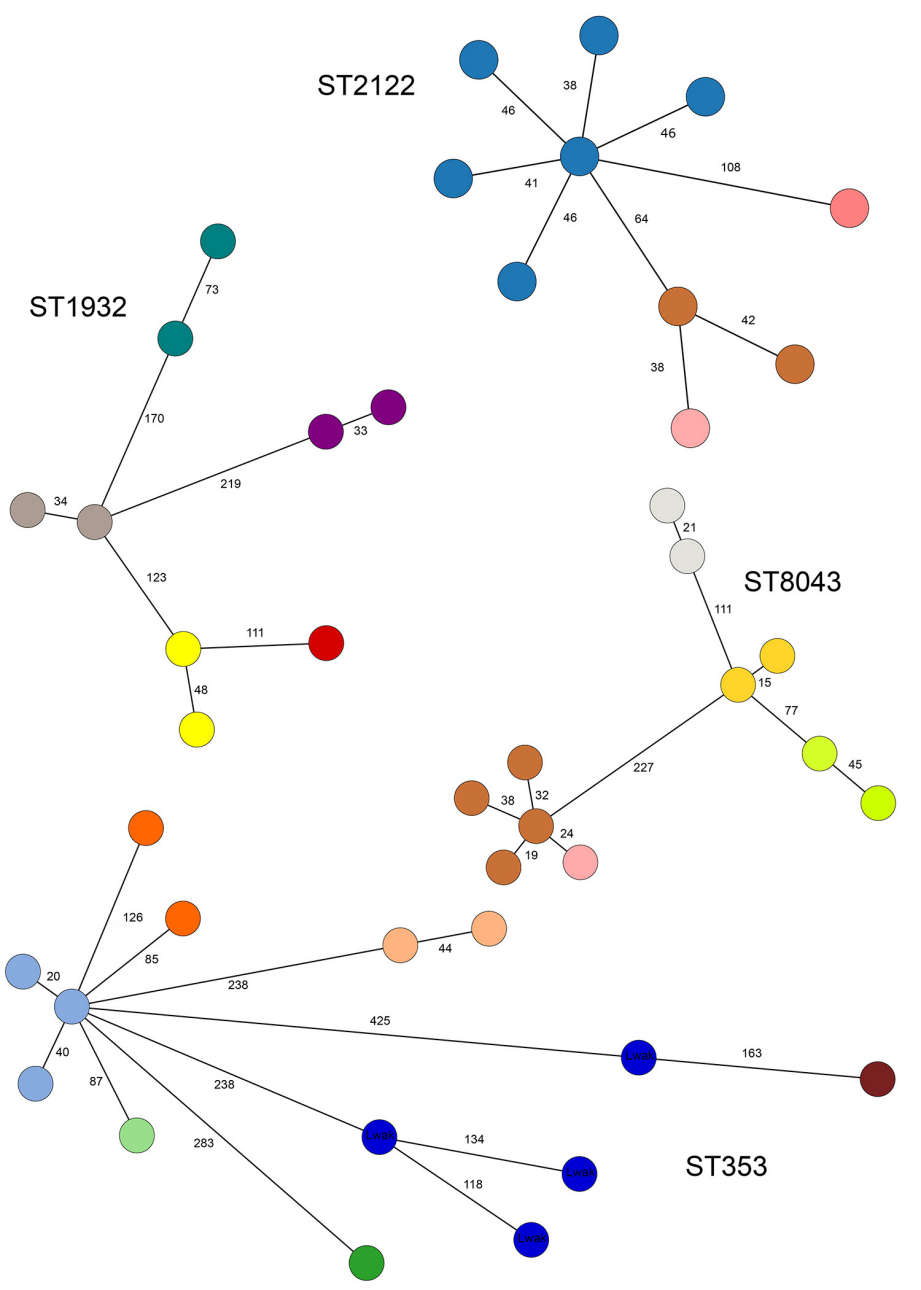
Minimum spanning tree of core-genome multilocus sequence types profiles from the 4 most prevalent 7-gene multilocus sequence type–based ST isolated from poultry in Tanzania, 2016–2017, in a study of *Campylobacter jejuni* and *C. coli,* East Africa. Each color represents a different farm, with the exception of 4 ST353 isolates from human cases (dark blue). Core genome multilocus sequence type allele differences are indicated on each branch. The 2 pairs of isolates from different farms with the lowest number of allele differences, belonging to ST8043 (24 allele differences) and ST2122 (38 allele differences) (shown in brown and light pink) were from 2 farms in the same location in Luongo, Moshi. One was an intensive indigenous farm (light pink isolates), and the other was an intensive broiler farm (brown isolates). ST, sequence type.

Of the 29 STs from poultry samples, 9 (31%) were identified on multiple farms (e.g., ST353 on 6 [9.1%] farms, ST1932 and ST8043 on 5 [7.6%] farms each, and ST2122 on 4 [4.5%] farms) (Figure 3). ST353, ST1932, and ST2122 were identified in Arusha and Moshi, whereas ST8043 was only identified in Moshi. For isolates belonging to the same ST, the median pairwise allele difference between isolates from the same farm on the basis of cgMLST was 46 (interquartile range [IQR] 38–59.75, range 15–230), whereas the median pairwise allele difference between isolates from different farms was 167 (IQR 102.5–245, range 24–497).

### Antimicrobial Resistance

AMR was detected in 75.3% (95% CrI 65.4–83.9%) and MDR was detected in 2.5% (95% CrI 0.3–6.8%) of 81 human isolates, and the point estimates of prevalence were similar in both regions (75.7% in Kibera and 75.0% in Lwak for AMR and 2.7% in Kibera and 2.3% in Lwak for MDR). No evidence of a trend in resistance in human isolates over the period of collection was seen. In poultry, the crude estimate of AMR prevalence was 85.7% of 97 poultry isolates from 87.5% of 56 poultry samples. The crude estimate of MDR prevalence was 40.2% of 97 poultry isolates from 44.6% of 56 poultry samples. After allowing for clustering of isolates within farms, the estimated prevalence in poultry was 85.4% (95% CrI 70.6–95.8%) for AMR and 43.1% (95% CrI 25.6%–61.4%) for MDR. The estimated difference between poultry and humans was nonsignificant for AMR (10.1% [95% CrI −7.0% to 24.8%, including 0]), but significant for MDR (40.6% [95% CrI 22.7%–59.1%, excluding 0]).

The most resistant isolates were resistant to 5 of the 6 classes of antimicrobial drugs tested. That phenotype was observed in 5 isolates from 3 birds from 2 farms, an intensive indigenous farm and a broiler farm, and all were *C. coli* belonging to ST8043.

**Figure 4:**
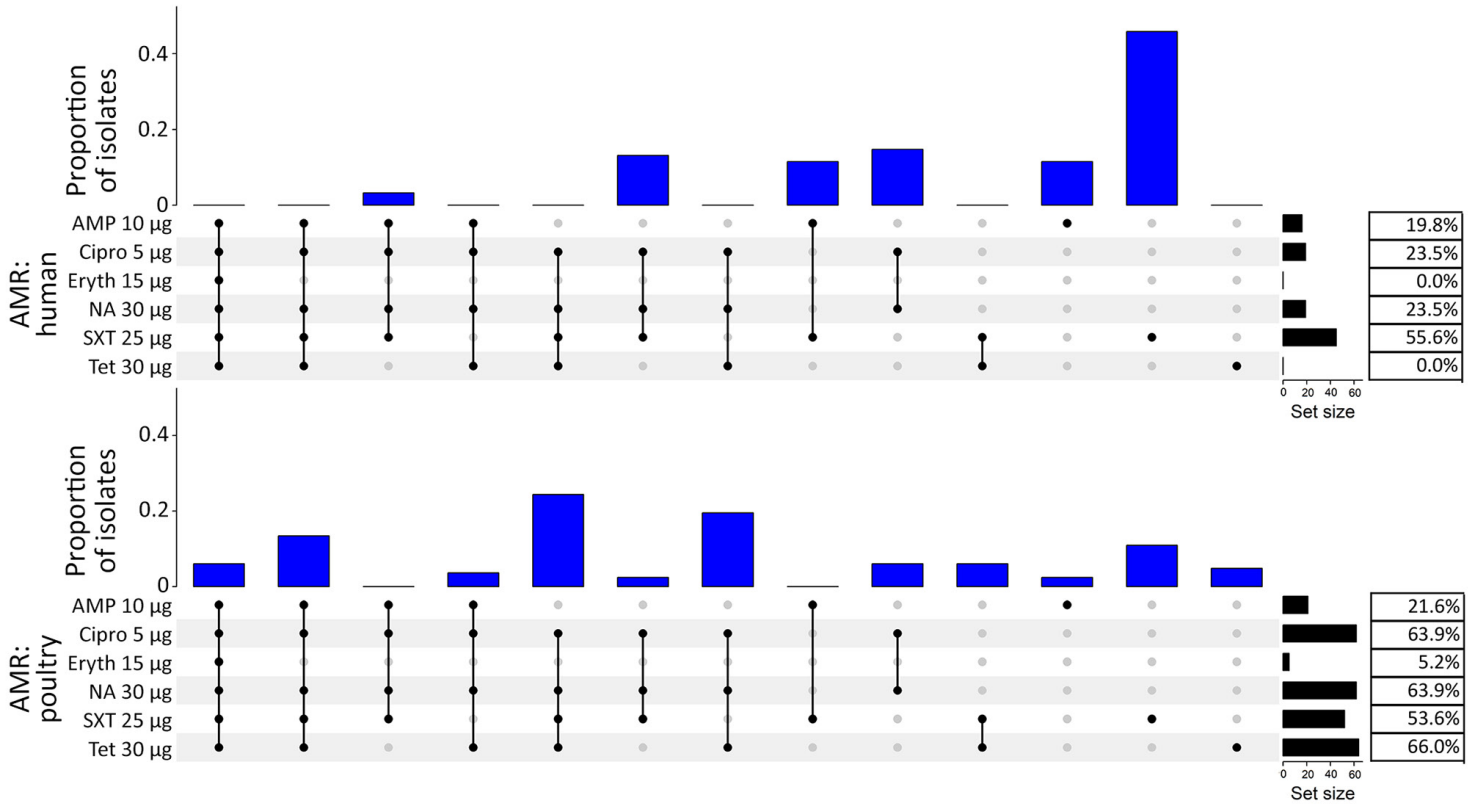
Distribution of different combinations of AMR in *Campylobacter jejuni* and *C. coli* human isolates from Kenya (top, n = 81) and poultry isolates from Tanzania (bottom, n = 97) in study of population structure and AMR in *C. jejuni* and *C. coli* isolated from humans with diarrhea and from poultry, East Africa, 2006–2017. The percentage of isolates resistant to each antimicrobial is given in the table to the right of each plot. All isolates were susceptible to gentamicin. The histogram represents proportion of isolates by antimicrobial resistance pattern. Black dots represent AMR and gray dots represent absence of AMR to the specific antimicrobial agent listed. Black lines join black dots to visualize patterns of AMR. AMP, ampicillin; AMR, antimicrobial resistance; Cipro, ciprofloxacin; Eryth, erythromycin; NA, nalidixic acid; SXT, trimethoprim/sulfamethoxazole; Tet, tetracycline.

**Figure 5:**
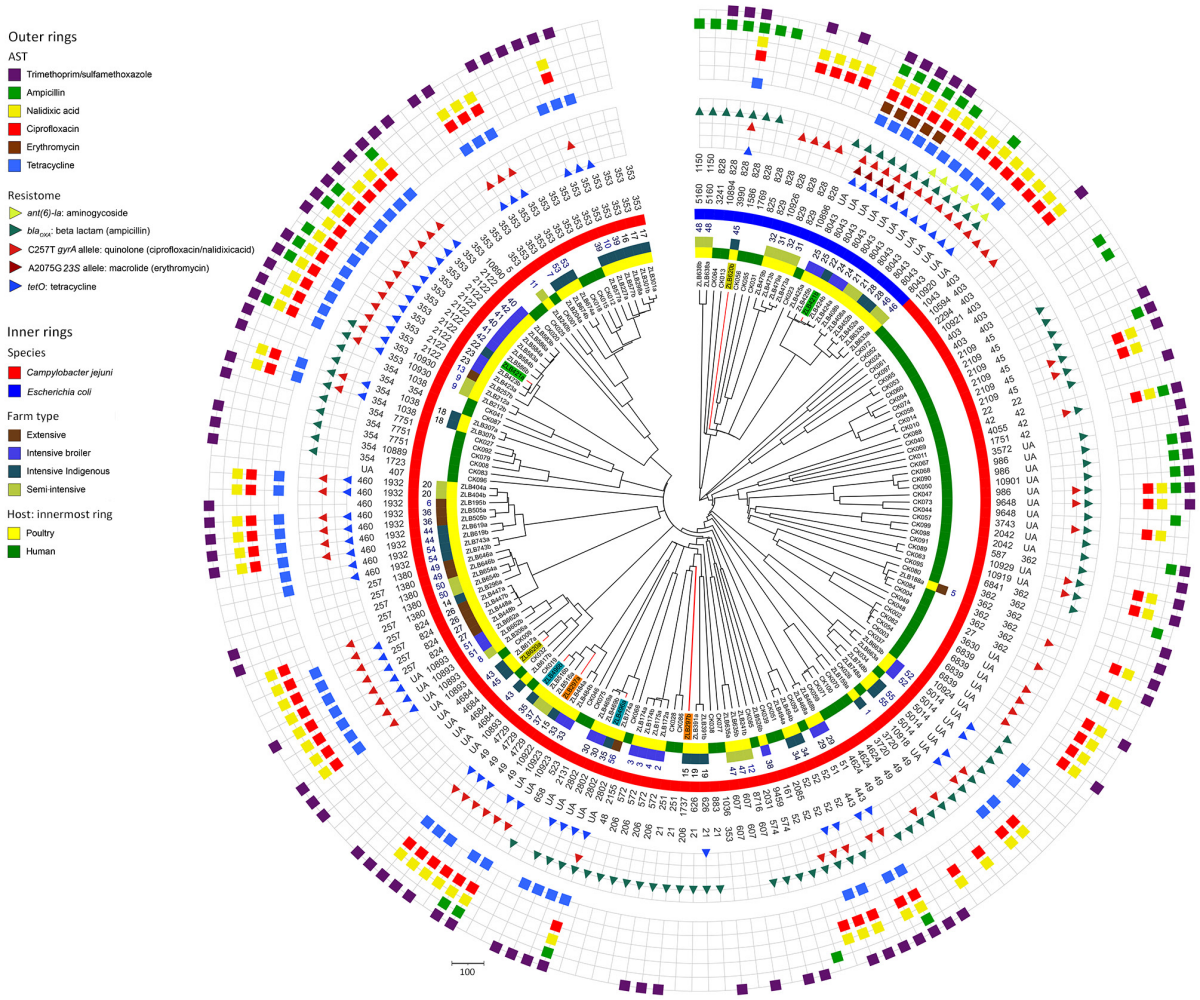
Circular dendrogram showing, from outer to inner rings, AST in study of population structure and antimicrobial resistance in *Campylobacter jejuni* and *C. coli* isolated from humans with diarrhea and from poultry, East Africa, 2006–2017. Colored blocks indicate resistance (all isolates were susceptible to gentamicin so this ring is not included), resistome, clonal complex (CC), sequence type (ST), *Campylobacter* species, poultry sample number, farm type, host and isolate ID for isolates from Kenya and Tanzania, 2006–2017 (human) or 2016–2017 (poultry). Isolates from the same poultry sample that belonged to a different ST are highlighted (samples 15, 22, 35, and 45) using colored isolate identification labels. The resistome indicates detection of resistance genes (encoding for resistance to some aminoglycosides, β-lactam antimicrobials and tetracyclines) and alleles (encoding for resistance for fluoroquinolones in the gyrase A gene, and macrolides in the 23S rRNA gene). Clustering of antimicrobial resistance phenotypes and the resistome with some CCs and STs is evident. For example, multidrug resistance is observed in *C. jejuni* ST2122 and *C. coli* ST8043 isolates. Scale bar indicates number of core-genome multilocus-sequence typing allele differences. AST, antimicrobial sensitivity.

The distribution of AST profiles differed between human and poultry isolates; 6 of the 9 MDR profiles were only found in poultry, and of the 5 most common resistance profiles among poultry isolates, only 1 was also detected in human isolates (Figure 4). The most common AMR profile in human isolates was resistance to trimethoprim/sulfamethoxazole, whereas the most common profile in poultry isolates was resistance to ciprofloxacin, nalidixic acid, trimethoprim/sulfamethoxazole, and tetracycline. Of the 41 birds with 2 isolates, 36 (87.8%) had pairs of isolates with identical AST profiles (Figure 5). All isolates were susceptible to gentamicin.

### Relationship among AST, Genotype, and Host

The relationship among the population structure, as determined by single linkage clustering of cgMLST profiles, and other epidemiologic, genotypic, and phenotypic variables was displayed as a circular dendrogram (Figure 5). We observed near-complete concordance between the genomically derived resistome and AST phenotypes associated with fluoroquinolone, macrolide, and tetracycline resistance. All isolates that were resistant to ciprofloxacin and nalidixic acid carried the C257T mutation, associated with fluoroquinolone resistance in the *gyr*A gene. Similarly, we observed complete concordance between the presence of the A2075G mutation in the 23S rRNA gene, associated with macrolide resistance and resistance to erythromycin. Tetracycline resistance was found in all isolates with the *tet*O gene, with the exception of isolate ZLB391b. In contrast, agreement was relatively poor between the presence of the β-lactamase–encoding gene *bla*_OXA_ and ampicillin resistance, and no isolates with genes associated with aminoglycoside resistance were resistant to gentamicin.

## Discussion

This study provides a detailed description of the population structure of *Campylobacter* spp. isolated from clinically healthy poultry and persons with diarrhea in East Africa and associations with AMR phenotypes, genes, and alleles. Key findings include evidence of a relatively high prevalence of AMR (>75%) in both human and poultry isolates and a higher prevalence of MDR in isolates from poultry than in those from humans. Further, considerable genetic heterogeneity within and between human and poultry *Campylobacter* isolates and many previously unreported STs were observed. The absence of dominant STs is in contrast to findings for nontyphoidal *Salmonella* collected from the same region over the same time period where 2 STs include almost two thirds of human diarrhea–derived isolates and 4 STs account for more than three quarters of poultry-derived isolates (*19*).

Earlier studies conducted in Tanzania estimated a similarly high farm-level prevalence of *Campylobacter* in poultry and also provided evidence of a higher prevalence of *C. jejuni* than *C. coli* and a higher prevalence in free-range chicken than in broilers (*10*,*33*). Those Tanzania studies did not examine AMR or sequence data. In contrast, a study conducted in Botswana showed evidence of a relatively higher prevalence of *C. coli* than *C. jejuni* in broilers compared with free-range poultry (*16*), and a higher prevalence of AMR was observed in *C. coli* than in *C. jejuni*. In common with our Tanzania study, the Botswana study used WGS and included 3 isolates belonging to *C. coli* ST8043, all of which carried *tetO* and *bla*_OXA_ genes; 1 had the *gyrA* C257T mutation encoding for quinolone resistance. Both in our study and in the study from Botswana, some STs were shared between humans and poultry, suggesting the possibility of interspecies transmission as also observed for certain types of nontyphoidal *Salmonella* (*19*). However, both studies were population-level studies, and analysis of epidemiologically linked isolates (e.g., from humans and animals within the same household or farm, or from poultry meat and its handlers and consumers) would be needed to generate direct evidence of interspecies transmission.

Comparison of the cgMLST allele differences within and between poultry farms showed greater similarity of isolates within farms compared with between farms, consistent with lower between-farm transmission than within-farm transmission. However, the 2 pairs of isolates from different farms with the lowest number of allele differences, belonging to ST8043 (24 allele differences) and ST2122 (38 allele differences) (Figure 3), were from 2 farms in the same location in Luongo, Moshi. Of those farms, 1 was an intensive indigenous farm and the other an intensive broiler farm. This finding is consistent with local spread or spread from a common source, such as shared equipment, inputs, or the environment (*34*), underlining the importance of biosecurity for preventing the spread of foodborne pathogens and AMR within the poultry sector.

Other studies of potential sources of human campylobacteriosis in Tanzania include *C. jejuni* and *C. coli* in duck intestinal contents (*35*), pig fecal samples (*36*), and beef carcasses and raw milk (*37*). In the study of beef carcasses and raw milk, prevalence of AMR was similar to that observed in poultry in our study.

Few studies have reported MDR prevalence estimates in human isolates in Kenya. However, 1 review (*6*) indicates a low prevalence of AMR in human isolates across multiple antimicrobials, similar to other countries in Africa and this study. By inference from individual AMR prevalence estimates, MDR in *C. jejuni* in Kenya was at most ≈10% (*6*).

Of the 78 STs identified in this study, 27 have been isolated from other countries in Africa and recorded on PubMLST. Those isolates include the most prevalent STs: ST353, which has also been isolated from Malawi, and ST1932 and ST8043, which have been isolated from Botswana (Appendix Table 2). 

With the exception of β-lactams and aminoglycosides, the resistome was strongly correlated with the AST results; the *tetO* gene was associated with tetraycline resistance (*38*), the C257T mutation in *gyrA* was associated with fluoroquinolone resistance (*39*), and the A2075G mutation in the 23S rRNA was associated with macrolide resistance (*40*). The A2075G mutation in the 23S rRNA (*E. coli* equivalent base 2058) was observed only in *C. coli* isolates in this study, which is consistent with other international studies (*40*). The relatively high prevalence of tetracycline and fluoroquinolone resistance in human or poultry isolates might be the result of selection pressure resulting from the widespread use of these antimicrobials in humans, food production, or both. A recent study in Dar es Salaam, Tanzania, indicated widespread use of both tetracyclines and fluoroquinolones in poultry and cattle production; >40% of farmers surveyed were not compliant with drug withdrawal periods (*41*). Evidence of noncompliance with withdrawal periods was also reported in a study of commercial smallholder egg producers in Morogoro, Tanzania (*42*). In human medicine, evidence exists of wide availability and sale of fluoroquinolones in authorized and unauthorized drug outlets. More than 70% of pharmacists surveyed reported dispensing antimicrobial drugs without a prescription, including penicillins, macrolides, and fluoroquinolones (*43*).

Our study had relatively low power to detect associations between epidemiologic variables and AMR and did not assess all transmission pathways. In addition, human diarrheal isolates were sourced from a wider temporal range (2006–2016) than poultry isolates (2016–2017), and isolates were co-located at regional rather than village or household level. However, although the human isolates were from a different country to the poultry isolates, it is worth noting that Nairobi is 272 km by road from Arusha and 326 km from Moshi, closer than other locations in Tanzania with human *Campylobacter* isolates potentially available for sequencing. Chicken production systems are largely similar across East Africa, including flock sizes and extensive, semi-intensive and intensive management systems between Kenya and Tanzania (*44*).

In conclusion, this study provides a detailed examination of the population structure of isolates of *C. jejuni* and *C. coli* in a region of East Africa. Although this study was smaller than similar studies conducted in high-income countries (*13*), it is one of the largest studies using WGS to characterize *Campylobacter* spp. isolates in Africa and has generated several valuable insights. The study showed a striking diversity of *Campylobacter* in both humans and poultry, with some STs common to multiple farms or to humans and poultry. AMR was highly prevalent, particularly to tetracyclines, fluoroquinolones, or sulphonamides, and MDR was prevalent in a high proportion of poultry compared with human isolates. The high prevalence of MDR and the identification of previously undescribed STs highlights the need for ongoing investigation of enteric pathogens, such as *Campylobacter* spp., in low-resource settings. That effort would require genomic tools to be embedded within formal and transparent surveillance systems, in addition to a greater understanding of the role of antimicrobial use and biosecurity measures as drivers of the emergence of resistance in human health and food production and improved governance of antimicrobial use in both sectors.

Appendix Additional information about population structure and antimicrobial resistance in *Campylobacter jejuni* and *C. coli* isolated from humans with diarrhea and from poultry, East Africa, 2006–2017.
