# Supplementary material for: Population Structure and Antimicrobial Resistance in Campylobacter jejuni and C. coli Isolated from Humans with Diarrhea and from Poultry, East Africa
Source: Emerg Infect Dis. 2024 Oct;30(10):2079–89. doi: 10.3201/eid3010.231399 (PMC11431929; doi:10.3201/eid3010.231399)
Supplement: Appendix — Additional information about population structure and antimicrobial resistance in Campylobacter jejuni and C. coli isolated from humans with diarrhea and from poultry, East Africa, 2006–2017. [file 23-1399-Techapp-s1.pdf]

Article DOI: https://doi.org/10.3201/eid3010.231399

EID cannot ensure accessibility for supplementary materials supplied by authors. Readers who have difficulty accessing supplementary content should contact the authors for assistance.

Population Structure and Antimicrobial Resistance in *Campylobacter jejuni* and *C. coli* Isolated from Humans with Diarrhea and from Poultry, East Africa

Appendix

Further details of the methods used for culture and isolation.

For poultry samples swab tips were enriched in 20mL Bolton broth, supplemented with 5% horse blood (TCS Biosciences, Botolph Claydon, UK) and selective supplement SR0208E (Oxoid, Basingstoke, UK). After 48 h incubation in a micro-aerophilic atmosphere (CampyGen; Oxoid), enrichments were plated on modified charcoal cefoperazone deoxycholate agar (mCCDA; Oxoid) and incubated for 48 h micro-aerophilically.

For human samples whole stool samples were swabbed and transported to the Enterics laboratory in Caryblair Transport media. In the lab, the swabs were inoculated on Campylobacter agar medium plates (CM0935, Oxoid) with supplement (SR0167, Oxoid) using standard microbiology procedures and incubated at 42o C for 48 hours under microaerophilic conditions. Colonies with typical morphology of Campylobacter were examined microscopically and confirmed biochemically by oxidase and hippurate hydrolysis tests.

Appendix Table 1. Isolate details showing the sample date, *Campylobacter* species, host, 7-gene MLST Sequence Type (ST) and Clonal Complex (CC), poultry sample number, poultry farm type and location. UA indicates the ST has not been assigned to a Clonal Complex.

ID no.	Sample date	species	Host	ST	CC	Poultry sample no.	Poultry farm type	Location	Accession
CK001	11/05/2011	*C. jejuni*	Human	353	353	NA	NA	Lwak	SRX22036608
CK002	29/05/2012	*C. jejuni*	Human	6839	UA	NA	NA	Lwak	SRX22036609
CK003	19/08/2011	*C. jejuni*	Human	6839	UA	NA	NA	Lwak	SRX22036762
CK004	9/08/2011	*C. jejuni*	Human	362	362	NA	NA	Lwak	SRX22036613
CK008	14/11/2013	*C. jejuni*	Human	10889	354	NA	NA	Lwak	SRX22036624
CK009	20/11/2013	*C. jejuni*	Human	10893	UA	NA	NA	Lwak	SRX22036635
CK010	10/10/2013	*C. jejuni*	Human	2109	45	NA	NA	Lwak	SRX22036710
CK011	17/02/2014	*C. jejuni*	Human	1751	42	NA	NA	Lwak	SRX22036721
CK012	10/03/2014	*C. jejuni*	Human	353	353	NA	NA	Lwak	SRX22036732
CK013	17/03/2014	*C. coli*	Human	10894	828	NA	NA	Lwak	SRX22036775
CK014	18/03/2014	*C. jejuni*	Human	2109	45	NA	NA	Lwak	SRX22036610
CK015	27/11/2014	*C. jejuni*	Human	353	353	NA	NA	Lwak	SRX22036653
CK018	14/04/2010	*C. jejuni*	Human	353	353	NA	NA	Lwak	SRX22036664
CK019	27/01/2016	*C. jejuni*	Human	10893	UA	NA	NA	Lwak	SRX22036675
CK020	14/09/2015	*C. jejuni*	Human	10890	353	NA	NA	Lwak	SRX22036686
CK023	10/06/2014	*C. coli*	Human	10896	828	NA	NA	Lwak	SRX22036697
CK024	7/11/2014	*C. jejuni*	Human	10594	403	NA	NA	Lwak	SRX22036740
CK025	27/11/2012	*C. jejuni*	Human	5	353	NA	NA	Lwak	SRX22036751
CK026	16/01/2014	*C. jejuni*	Human	10918	UA	NA	NA	Lwak	SRX22036760
CK027	6/12/2013	*C. jejuni*	Human	7751	354	NA	NA	Lwak	SRX22036761
CK028	26/11/2012	*C. jejuni*	Human	251	21	NA	NA	Lwak	SRX22036763
CK031	6/11/2012	*C. coli*	Human	825	828	NA	NA	Lwak	SRX22036764
CK032	6/06/2012	*C. jejuni*	Human	4684	UA	NA	NA	Lwak	SRX22036765
CK034	23/10/2014	*C. jejuni*	Human	5014	UA	NA	NA	Lwak	SRX22036766
CK037	21/01/2016	*C. jejuni*	Human	10924	UA	NA	NA	Lwak	SRX22036767
CK038	22/01/2016	*C. jejuni*	Human	883	21	NA	NA	Lwak	SRX22036768
CK039	21/03/2016	*C. jejuni*	Human	161	52	NA	NA	Lwak	SRX22036769
CK040	19/10/2012	*C. jejuni*	Human	42	42	NA	NA	Lwak	SRX22036770
CK041	22/03/2016	*C. jejuni*	Human	1038	354	NA	NA	Lwak	SRX22036771
CK044	27/06/2013	*C. jejuni*	Human	9648	UA	NA	NA	Lwak	SRX22036612
CK046	1/12/2014	*C. jejuni*	Human	523	658	NA	NA	Lwak	SRX22036614
CK047	9/12/2013	*C. jejuni*	Human	986	UA	NA	NA	Kibera	SRX22036615
CK048	15/01/2014	*C. jejuni*	Human	3630	UA	NA	NA	Kibera	SRX22036616
CK049	5/05/2014	*C. jejuni*	Human	27	362	NA	NA	Kibera	SRX22036617
CK050	3/06/2014	*C. jejuni*	Human	10901	UA	NA	NA	Kibera	SRX22036618
CK051	3/06/2014	*C. jejuni*	Human	2085	52	NA	NA	Kibera	SRX22036619
CK052	24/05/2014	*C. jejuni*	Human	1043	403	NA	NA	Kibera	SRX22036620
CK053	7/05/2008	*C. jejuni*	Human	403	403	NA	NA	Kibera	SRX22036621
CK054	24/07/2008	*C. jejuni*	Human	6839	UA	NA	NA	Kibera	SRX22036622
CK055	14/01/2011	*C. coli*	Human	1769	828	NA	NA	Kibera	SRX22036623
CK056	8/09/2010	*C. coli*	Human	1586	828	NA	NA	Lwak	SRX22036625
CK057	3/07/2007	*C. jejuni*	Human	3743	UA	NA	NA	Kibera	SRX22036626
CK058	10/03/2009	*C. jejuni*	Human	2109	45	NA	NA	Kibera	SRX22036627
CK059	30/01/2009	*C. jejuni*	Human	4624	49	NA	NA	Kibera	SRX22036628
CK060		*C. jejuni*	Human	2109	45	NA	NA	Kibera	SRX22036629
CK061	20/06/2008	*C. jejuni*	Human	2294	403	NA	NA	Kibera	SRX22036630
CK063	19/11/2008	*C. jejuni*	Human	10919	UA	NA	NA	Kibera	SRX22036631
CK064	27/01/2009	*C. coli*	Human	3241	828	NA	NA	Kibera	SRX22036632
CK065	6/02/2009	*C. jejuni*	Human	403	403	NA	NA	Kibera	SRX22036633
CK066	14/01/2009	*C. jejuni*	Human	2155	48	NA	NA	Lwak	SRX22036634
CK067	8/07/2008	*C. jejuni*	Human	3572	UA	NA	NA	Kibera	SRX22036636
CK068	27/09/2008	*C. jejuni*	Human	986	UA	NA	NA	Kibera	SRX22036637
CK069	1/08/2008	*C. jejuni*	Human	4055	42	NA	NA	Lwak	SRX22036638
CK070	11/11/2008	*C. jejuni*	Human	3720	49	NA	NA	Kibera	SRX22036639
CK071	18/03/2009	*C. jejuni*	Human	4624	49	NA	NA	Kibera	SRX22036640
CK072	18/03/2009	*C. jejuni*	Human	10920	UA	NA	NA	Kibera	SRX22036641
CK073	11/03/2008	*C. jejuni*	Human	9648	UA	NA	NA	Kibera	SRX22036642
CK074	21/01/2009	*C. jejuni*	Human	2109	45	NA	NA	Kibera	SRX22036643
CK075	3/03/2009	*C. jejuni*	Human	2131	UA	NA	NA	Kibera	SRX22036708
CK077	21/03/2011	*C. jejuni*	Human	1036	353	NA	NA	Lwak	SRX22036709
CK079	11/08/2010	*C. jejuni*	Human	7751	354	NA	NA	Lwak	SRX22036711

ID no.	Sample date	species	Host	ST	CC	Poultry sample no.	Poultry farm type	Location	Accession
CK080	6/08/2010	*C. jejuni*	Human	362	362	NA	NA	Lwak	SRX22036712
CK082	16/03/2011	*C. jejuni*	Human	6839	UA	NA	NA	Lwak	SRX22036713
CK083		*C. jejuni*	Human	1723	354	NA	NA	Kibera	SRX22036714
CK084	5/01/2011	*C. jejuni*	Human	362	362	NA	NA	Lwak	SRX22036715
CK085	8/06/2010	*C. jejuni*	Human	2031	574	NA	NA	Lwak	SRX22036716
CK086	18/02/2011	*C. jejuni*	Human	251	21	NA	NA	Lwak	SRX22036717
CK087	8/06/2010	*C. jejuni*	Human	354	354	NA	NA	Lwak	SRX22036718
CK088	22/06/2010	*C. jejuni*	Human	22	22	NA	NA	Lwak	SRX22036719
CK089	1/12/2008	*C. jejuni*	Human	10929	UA	NA	NA	Kibera	SRX22036720
CK090	17/09/2008	*C. jejuni*	Human	986	UA	NA	NA	Kibera	SRX22036722
CK091	25/11/2008	*C. jejuni*	Human	587	362	NA	NA	Kibera	SRX22036723
CK092	10/03/2009	*C. jejuni*	Human	7751	354	NA	NA	Lwak	SRX22036724
CK093	14/12/2006	*C. jejuni*	Human	52	52	NA	NA	Kibera	SRX22036725
CK094	16/02/2007	*C. jejuni*	Human	2109	45	NA	NA	Kibera	SRX22036726
CK095	14/03/2009	*C. jejuni*	Human	6841	362	NA	NA	Kibera	SRX22036727
CK096	13/03/2009	*C. jejuni*	Human	407	UA	NA	NA	Kibera	SRX22036728
CK097	26/01/2010	*C. jejuni*	Human	10921	403	NA	NA	Kibera	SRX22036729
CK098	8/10/2008	*C. jejuni*	Human	2042	UA	NA	NA	Kibera	SRX22036730
CK099	24/05/2007	*C. jejuni*	Human	2042	UA	NA	NA	Kibera	SRX22036731
CK100	16/10/2008	*C. jejuni*	Human	4624	49	NA	NA	Kibera	SRX22036733
ZLB159a	10/10/2016	*C. jejuni*	Poultry	3720	49	1	Intensive Indigenous	Arusha	SRX22036734
ZLB172a	10/10/2016	*C. jejuni*	Poultry	572	206	2	Intensive broiler	Arusha	SRX22036735
ZLB174a	10/10/2016	*C. jejuni*	Poultry	572	206	3	Intensive broiler	Arusha	SRX22036736
ZLB174b	10/10/2016	*C. jejuni*	Poultry	572	206	3	Intensive broiler	Arusha	SRX22036737
ZLB175a	10/10/2016	*C. jejuni*	Poultry	572	206	4	Intensive broiler	Arusha	SRX22036738
ZLB188a	17/10/2016	*C. jejuni*	Poultry	362	362	5	Extensive	Arusha	SRX22036739
ZLB195b	17/10/2016	*C. jejuni*	Poultry	1932	460	6	Extensive	Arusha	SRX22036772
ZLB204a	17/10/2016	*C. jejuni*	Poultry	353	353	7	Intensive Indigenous	Arusha	SRX22036773
ZLB206a	17/10/2016	*C. jejuni*	Poultry	10893	UA	8	Semi intensive	Arusha	SRX22036774
ZLB212a	17/10/2016	*C. jejuni*	Poultry	10930	354	9	Semi intensive	Arusha	SRX22036776
ZLB212b	17/10/2016	*C. jejuni*	Poultry	10930	354	9	Semi intensive	Arusha	SRX22036777
ZLB227a	24/10/2016	*C. jejuni*	Poultry	353	353	10	Intensive Indigenous	Arusha	SRX22036778
ZLB240b	24/10/2016	*C. jejuni*	Poultry	353	353	11	Semi intensive	Arusha	SRX22036779
ZLB241b	24/10/2016	*C. jejuni*	Poultry	8716	607	12	Semi intensive	Arusha	SRX22036780
ZLB257b	24/10/2016	*C. jejuni*	Poultry	2122	353	13	Extensive	Arusha	SRX22036781
ZLB296a	14/11/2016	*C. jejuni*	Poultry	1380	257	14	Intensive Indigenous	Arusha	SRX22036782
ZLB297a	14/11/2016	*C. jejuni*	Poultry	10922	49	15	Intensive Indigenous	Arusha	SRX22036783
ZLB297b	14/11/2016	*C. jejuni*	Poultry	1737	206	15	Intensive Indigenous	Arusha	SRX22036784
ZLB298a	14/11/2016	*C. jejuni*	Poultry	353	353	16	Intensive Indigenous	Arusha	SRX22036785
ZLB301a	14/11/2016	*C. jejuni*	Poultry	353	353	17	Intensive Indigenous	Arusha	SRX22036611
ZLB301b	14/11/2016	*C. jejuni*	Poultry	353	353	17	Intensive Indigenous	Arusha	SRX22036644
ZLB307a	21/11/2016	*C. jejuni*	Poultry	1038	354	18	Intensive Indigenous	Arusha	SRX22036645
ZLB307b	21/11/2016	*C. jejuni*	Poultry	1038	354	18	Intensive Indigenous	Arusha	SRX22036646
ZLB391a	5/12/2016	*C. jejuni*	Poultry	626	21	19	Intensive Indigenous	Arusha	SRX22036647
ZLB391b	5/12/2016	*C. jejuni*	Poultry	626	21	19	Intensive Indigenous	Arusha	SRX22036648
ZLB404a	9/04/2017	*C. jejuni*	Poultry	1932	460	20	Semi intensive	Moshi	SRX22036649
ZLB404b	9/04/2017	*C. jejuni*	Poultry	1932	460	20	Semi intensive	Moshi	SRX22036650
ZLB408a	9/04/2017	*C. coli*	Poultry	8043	UA	21	Semi intensive	Moshi	SRX22036651
ZLB408b	9/04/2017	*C. coli*	Poultry	8043	UA	21	Semi intensive	Moshi	SRX22036652
ZLB421a	9/04/2017	*C. jejuni*	Poultry	2122	353	22	Intensive Indigenous	Moshi	SRX22036654

ID no.	Sample date	species	Host	ST	CC	Poultry sample no.	Poultry farm type	Location	Accession
ZLB421b	9/04/2017	*C. coli*	Poultry	8043	UA	22	Intensive Indigenous	Moshi	SRX22036655
ZLB423a	9/04/2017	*C. jejuni*	Poultry	2122	353	23	Intensive broiler	Moshi	SRX22036656
ZLB423b	9/04/2017	*C. jejuni*	Poultry	2122	353	23	Intensive broiler	Moshi	SRX22036657
ZLB424a	9/04/2017	*C. coli*	Poultry	8043	UA	24	Intensive broiler	Moshi	SRX22036658
ZLB424b	9/04/2017	*C. coli*	Poultry	8043	UA	24	Intensive broiler	Moshi	SRX22036659
ZLB425a	9/04/2017	*C. coli*	Poultry	8043	UA	25	Intensive broiler	Moshi	SRX22036660
ZLB425b	9/04/2017	*C. coli*	Poultry	8043	UA	25	Intensive broiler	Moshi	SRX22036661
ZLB447a	15/05/2017	*C. jejuni*	Poultry	824	257	26	Extensive	Moshi	SRX22036662
ZLB447b	15/05/2017	*C. jejuni*	Poultry	824	257	26	Extensive	Moshi	SRX22036663
ZLB448a	15/05/2017	*C. jejuni*	Poultry	824	257	27	Extensive	Moshi	SRX22036665
ZLB448b	15/05/2017	*C. jejuni*	Poultry	824	257	27	Extensive	Moshi	SRX22036666
ZLB452a	15/05/2017	*C. coli*	Poultry	8043	UA	28	Intensive Indigenous	Moshi	SRX22036667
ZLB452b	15/05/2017	*C. coli*	Poultry	8043	UA	28	Intensive Indigenous	Moshi	SRX22036668
ZLB468a	15/05/2017	*C. jejuni*	Poultry	51	443	29	Intensive broiler	Moshi	SRX22036669
ZLB468b	15/05/2017	*C. jejuni*	Poultry	51	443	29	Intensive broiler	Moshi	SRX22036670
ZLB469a	15/05/2017	*C. jejuni*	Poultry	2802	UA	30	Intensive broiler	Moshi	SRX22036671
ZLB469b	15/05/2017	*C. jejuni*	Poultry	2802	UA	30	Intensive broiler	Moshi	SRX22036672
ZLB473a	15/05/2017	*C. coli*	Poultry	829	828	31	Semi intensive	Moshi	SRX22036673
ZLB473b	15/05/2017	*C. coli*	Poultry	10926	828	31	Semi intensive	Moshi	SRX22036674
ZLB479a	15/05/2017	*C. coli*	Poultry	829	828	32	Semi intensive	Moshi	SRX22036676
ZLB479b	15/05/2017	*C. coli*	Poultry	829	828	32	Semi intensive	Moshi	SRX22036677
ZLB484a	22/05/2017	*C. jejuni*	Poultry	10923	UA	33	Intensive broiler	Moshi	SRX22036678
ZLB484b	22/05/2017	*C. jejuni*	Poultry	10923	UA	33	Intensive broiler	Moshi	SRX22036679
ZLB494a	22/05/2017	*C. jejuni*	Poultry	52	52	34	Intensive Indigenous	Moshi	SRX22036680
ZLB494b	22/05/2017	*C. jejuni*	Poultry	52	52	34	Intensive Indigenous	Moshi	SRX22036681
ZLB496a	22/05/2017	*C. jejuni*	Poultry	2802	UA	35	Intensive Indigenous	Moshi	SRX22036682
ZLB496b	22/05/2017	*C. jejuni*	Poultry	4729	49	35	Intensive Indigenous	Moshi	SRX22036683
ZLB505a	22/05/2017	*C. jejuni*	Poultry	1932	460	36	Extensive	Moshi	SRX22036684
ZLB505b	22/05/2017	*C. jejuni*	Poultry	1932	460	36	Extensive	Moshi	SRX22036685
ZLB516a	22/05/2017	*C. jejuni*	Poultry	4729	49	37	Semi intensive	Moshi	SRX22036687
ZLB516b	22/05/2017	*C. jejuni*	Poultry	4729	49	37	Semi intensive	Moshi	SRX22036688
ZLB526b	29/05/2017	*C. jejuni*	Poultry	9459	574	38	Intensive broiler	Moshi	SRX22036689
ZLB577a	22/06/2017	*C. jejuni*	Poultry	353	353	39	Intensive Indigenous	Moshi	SRX22036690
ZLB577b	22/06/2017	*C. jejuni*	Poultry	353	353	39	Intensive Indigenous	Moshi	SRX22036691
ZLB583a	22/06/2017	*C. jejuni*	Poultry	2122	353	40	Intensive broiler	Moshi	SRX22036692
ZLB583b	22/06/2017	*C. jejuni*	Poultry	2122	353	40	Intensive broiler	Moshi	SRX22036693
ZLB584a	22/06/2017	*C. jejuni*	Poultry	2122	353	41	Intensive broiler	Moshi	SRX22036694
ZLB584b	22/06/2017	*C. jejuni*	Poultry	2122	353	41	Intensive broiler	Moshi	SRX22036695
ZLB586a	22/06/2017	*C. jejuni*	Poultry	2122	353	42	Intensive broiler	Moshi	SRX22036696
ZLB586b	22/06/2017	*C. jejuni*	Poultry	2122	353	42	Intensive broiler	Moshi	SRX22036698
ZLB617a	28/06/2017	*C. jejuni*	Poultry	4684	UA	43	Intensive Indigenous	Moshi	SRX22036699
ZLB617b	28/06/2017	*C. jejuni*	Poultry	4684	UA	43	Intensive Indigenous	Moshi	SRX22036700
ZLB619a	28/06/2017	*C. jejuni*	Poultry	1932	460	44	Intensive Indigenous	Moshi	SRX22036701
ZLB619b	28/06/2017	*C. jejuni*	Poultry	1932	460	44	Intensive Indigenous	Moshi	SRX22036702
ZLB620a	28/06/2017	*C. jejuni*	Poultry	4684	UA	45	Intensive Indigenous	Moshi	SRX22036703
ZLB620b	28/06/2017	*C. coli*	Poultry	3990	828	45	Intensive Indigenous	Moshi	SRX22036704
ZLB633a	28/06/2017	*C. coli*	Poultry	8043	UA	46	Semi intensive	Moshi	SRX22036705
ZLB633b	28/06/2017	*C. coli*	Poultry	8043	UA	46	Semi intensive	Moshi	SRX22036706
ZLB635a	28/06/2017	*C. jejuni*	Poultry	607	607	47	Semi intensive	Moshi	SRX22036707
ZLB635b	28/06/2017	*C. jejuni*	Poultry	607	607	47	Semi intensive	Moshi	SRX22036741

ID no.	Sample date	species	Host	ST	CC	Poultry sample no.	Poultry farm type	Location	Accession
ZLB638a	28/06/2017	*C. coli*	Poultry	5160	1150	48	Semi intensive	Moshi	SRX22036742
ZLB638b	28/06/2017	*C. coli*	Poultry	5160	1150	48	Semi intensive	Moshi	SRX22036743
ZLB646a	3/07/2017	*C. jejuni*	Poultry	1380	257	49	Extensive	Moshi	SRX22036744
ZLB646b	3/07/2017	*C. jejuni*	Poultry	1380	257	49	Extensive	Moshi	SRX22036745
ZLB654a	3/07/2017	*C. jejuni*	Poultry	1380	257	50	Semi intensive	Moshi	SRX22036746
ZLB654b	3/07/2017	*C. jejuni*	Poultry	1380	257	50	Semi intensive	Moshi	SRX22036747
ZLB662a	3/07/2017	*C. jejuni*	Poultry	10893	UA	51	Intensive broiler	Moshi	SRX22036748
ZLB662b	3/07/2017	*C. jejuni*	Poultry	10893	UA	51	Intensive broiler	Moshi	SRX22036749
ZLB663a	3/07/2017	*C. jejuni*	Poultry	5014	UA	52	Intensive broiler	Moshi	SRX22036750
ZLB663b	3/07/2017	*C. jejuni*	Poultry	5014	UA	52	Intensive broiler	Moshi	SRX22036752
ZLB674a	3/07/2017	*C. jejuni*	Poultry	353	353	53	Intensive Indigenous	Moshi	SRX22036753
ZLB674b	3/07/2017	*C. jejuni*	Poultry	353	353	53	Intensive Indigenous	Moshi	SRX22036754
ZLB743a	17/07/2017	*C. jejuni*	Poultry	1932	460	54	Intensive Indigenous	Moshi	SRX22036755
ZLB743b	17/07/2017	*C. jejuni*	Poultry	1932	460	54	Intensive Indigenous	Moshi	SRX22036756
ZLB748a	17/07/2017	*C. jejuni*	Poultry	5014	UA	55	Intensive Indigenous	Moshi	SRX22036757
ZLB748b	17/07/2017	*C. jejuni*	Poultry	5014	UA	55	Intensive Indigenous	Moshi	SRX22036758
ZLB774a	24/07/2017	*C. jejuni*	Poultry	2802	UA	56	Extensive	Moshi	SRX22036759

Sequence data are available in BioProject PRJNA1026168.

Appendix Table 2. Table showing which of the sequence types (ST) identified in the present study have also been isolated from other countries in the African continent. Data were extracted from PubMLST (27) on 1 December 2021; numbers refer to the number of isolates.

	Kenya and Tanzania, this study			Africa, other studies							
ST	Human	Poultry	Total	Africa total	Botswana	Burkina Faso	Egypt	Malawi	Nigeria	Senegal	South Africa
5	1		1								
22	1		1	4				1		1	2
27	1		1								
42	1		1	1			1				
51		2	2	1	1						
52	1	2	3	7	3					4	
161	1		1								
251	2		2								
353	4	10	14	3				3			
354	1		1	6			4		2		
362	3	1	4	25		1		1			23
403	2		2								
407	1		1								
523	1		1	4		2			2		
572		4	4	2			2				
587	1		1								
607		2	2	3					3		
626		2	2								
824		4	4	2			1			1	
825	1		1	2			1		1		
829		3	3	3		3					
883	1		1	3	3						
986	3		3								
1036	1		1	14					3	11	
1038	1	2	3	4				1		3	
1043	1		1								
1380		5	5								
1586	1		1								
1723	1		1	3	3						
1737		1	1								
1751	1		1								

	Kenya and Tanzania, this study			Africa, other studies							
ST	Human	Poultry	Total	Africa total	Botswana	Burkina Faso	Egypt	Malawi	Nigeria	Senegal	South Africa
1769	1		1	1	1						
1932		9	9	7	1				6		
2031	1		1								
2042	2		2	2	1	1					
2085	1		1								
2109	6		6								
2122		10	10								
2131	1		1	3	3						
2155	1		1								
2294	1		1	1				1			
2802		4	4								
3241	1		1	4	3		1				
3572	1		1								
3630	1		1								
3720	1	1	2								
3743	1		1								
3990		1	1								
4055	1		1								
4624	3		3								
4684	1	3	4								
4729		3	3								
5014	1	4	5								
5160		2	2								
6839	4		4	1				1			
6841	1		1	1				1			
7751	3		3								
8043		11	11	3	3						
8716		1	1	2	2						
9459		1	1								
9648	2		2								
10594	1		1								
10889	1		1								
10890	1		1								
10893	2	3	5								
10894	1		1								
10896	1		1								
10901	1		1								
10918	1		1								
10919	1		1								
10920	1		1								
10921	1		1								
10922		1	1								
10923		2	2								
10924	1		1								
10926		1	1								
10929	1		1								
